# P-265. No Flu in the Loo: Efficacy of a novel, automated far ultraviolet-C light technology for decontamination of surfaces and aerosolized viral particles in bathrooms

**DOI:** 10.1093/ofid/ofae631.469

**Published:** 2025-01-29

**Authors:** Samir Memic, Claire Kaple, Jennifer Cadnum, Curtis Donskey

**Affiliations:** Northeast Ohio VA Healthcare System, Cleveland, Ohio; Case Western Reserve University, Cleveland, Ohio; Northeast Ohio VA Medical Center, Cleveland, Ohio; Cleveland VA Hospital, Cleveland, Ohio

## Abstract

**Background:**

Aerosols generated during toilet flushing are a potential source for transmission of

viral and bacterial pathogens in bathrooms. However, manual decontamination of bathrooms

after each use is not feasible.

Log 10 reduction of organisms on steel disk carriers after 2 hours of far UV-C light exposure*

**Figure:**
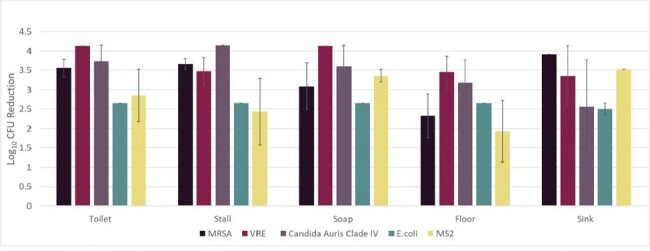

MRSA, methicillin-resistant Staphylococcus aureus; VRE, vancomycin-resistant enterococci; E. coli,

Escherichia coli, MS2, bacteriophage MS2

*, 5 of 9 test sites shown

**Methods:**

We tested the efficacy of a wall-mounted far ultraviolet-C (UV-C) light technology

that only delivers far UV-C when people are not present for decontamination of surfaces and

aerosolized viral particles in unoccupied hospital bathrooms. A quantitative disk carrier test

method was used to test efficacy against organisms on steel disk carriers placed in 9 sites in the

bathrooms with an exposure time of 2 hours; Clostridioides difficile spores were exposed for 24

hours. Efficacy against aerosolized bacteriophage MS2 was tested with a 45-minute exposure.

**Results:**

The far UV-C technology reduced methicillin-resistant Staphylococcus aureus

(MRSA), vancomycin-resistant enterococci (VRE), Candida auris, Escherichia coli, and

bacteriophage MS2 on disk carriers by >1.9 log 10 at all test sites in 2 hours (Figure), and reduced

C. difficile spores by >1.7 log 10 in 24 hours. Aerosolized bacteriophage MS2 was reduced by 4

log 10 plaque-forming units in 45 minutes. The technology consistently turned off when people

entered the bathroom and resumed far UV-C delivery after they exited.

**Conclusion:**

The novel far UV-C light technology could potentially be useful for automated

decontamination of bathrooms in healthcare and community settings.

**Disclosures:**

**Curtis Donskey, MD**, Clorox: Grant/Research Support|Pfizer: Grant/Research Support

